# Empowering future public health professionals: The impact of community-based education on students’ maternal and child health competencies

**DOI:** 10.1371/journal.pone.0345916

**Published:** 2026-06-01

**Authors:** Rubeena Zakar, Gulzar Shah, Ruhma Shahzad, Nazoora Manal Zakar, Ara Tekian

**Affiliations:** 1 Department of Public Health, University of the Punjab, Lahore, Punjab‌‌, Pakistan; 2 Department of Health Policy and Community Health, Jiann-Ping Hsu College of Public Health, Georgia Southern University, Statesboro, Georgia‌‌, United States of America; 3 Akhtar Saeed Medical and Dental College, Lahore, Punjab, Pakistan; 4 Department of Medical Education, University of Illinois Chicago, Chicago, Illinois, United States of America; Obafemi Awolowo University, NIGERIA

## Abstract

Preparing future public health professionals to address real-world maternal and child health (MCH) challenges requires educational approaches that extend beyond classroom instruction. In this context, integrating community-based education (CBE) into the curriculum serves as a pivotal strategy. This study assesses the implementation and effectiveness of a CBE module in MCH, designed for strengthening the knowledge and competencies of undergraduate public health students. A mixed-method pre- and post-intervention design was used to assess changes in students’ knowledge, skills, and practical competencies following one year of supervised community engagement in a semi-urban setting in Lahore, Pakistan. Quantitative assessments included survey questionnaires, quizzes, MCQs, and OSCE, while qualitative data were collected through focus group discussions, and self-reflection logs. Descriptive statistics, a paired-samples t-test, and a marginal homogeneity test were used to assess students’ learning and research outcomes. For qualitative data, thematic analysis was conducted using a deductive-inductive approach. Pre- and post- analysis revealed significant improvements (p < 0.001) in students’ knowledge across all MCH domains, along with enhanced skills of leadership, project/time management, communication, problem-solving, teamwork, handling unexpected situations, and designing community-tailored interventions (p < 0.001) following CBE implementation. Ongoing assessments indicated consistent learning with a mean quiz score of 80.2% (SD = 7.2), and a mean MCQ exam score of 81.5% (SD = 7.6). Qualitative data further revealed increased empathy, cultural sensitivity, and professional confidence. The study concludes that the CBE program provides students with valuable insights into the specific needs and challenges of communities related to MCH, thereby enhancing their knowledge and competency development. This experience supports the continuation of CBE programs for future students.

## Introduction

Higher education institutions are recognized as drivers of change within communities and society as a whole [1]. With the growing challenges of globalization, higher education has a greater responsibility to improve the educational system to meet real-world demands. This ensures that students gain theoretical knowledge, work in real-life settings, and contribute to empowering communities [2]. However, each community’s specific needs and problems may vary, creating ambiguity about the kind of world for which students should be prepared.

In this context, integrating community-based education (CBE) into the curriculum is a pivotal strategy, as it is an important learning approach in health sciences education [3]. CBE not only provides ‘real-world’ experience to students by involving them in learning activities that use the community as a learning environment, but also helps in improving the health indicators of communities where these CBE programs are implemented [4]. Additionally, students learn more about the range of social, political, and economic factors that affect health, known as the social determinants of health [5]. CBE has made significant contributions in developing countries, including Pakistan, where resources to cater to the health awareness needs of the communities are limited.

With a growing population that ranks Pakistan as the fifth most populous country globally, achieving sustainable development goals related to health, particularly maternal and child health (MCH), remains a formidable challenge. Maternal mortality ratio, standing at 186 per 100,000 live births, along with alarming rates of neonatal, infant, and child mortality, underscores the urgent need for robust MCH interventions [6]. These interventions must address a spectrum of needs, including prenatal care, neonatal health, maternal and infant mortality, immunization, nutrition, and access to specialized services.

In the Pakistani social setup, universities can play a significant role in improving community health through community outreach programs. Many studies provide evidence that community-academic partnerships can strengthen community awareness regarding health issues [7,8]. Community-based initiatives, such as behavior change interventions involving university students, can be beneficial in many ways. Several studies report improvements in MCH indicators, such as increased use of contraceptive methods, institutional deliveries, practicing skin-to-skin care, initiating timely and exclusively breastfeeding. Other indicators include providing age-appropriate immunization, identifying dangerous signs, and taking timely care in case of diarrhea and pneumonia in children under-five after introducing community-based interventions [6,9].

In Pakistan, a substantial portion of the healthcare budget is allocated to curative medicine, leaving public health as a relatively neglected area. Specifically, in Punjab, the country’s most populous province, only a few institutions offer degree programs in public health. Among these institutions, there is a notable absence of CBE programs focusing on Maternal, Newborn, and Child Health (MNCH). Additionally, the curriculum is classroom-based, and teachers lack experience and skills in organizing and teaching in the field. This gap underscores a critical need to integrate practical, community-focused training into public health curricula to better address the region’s specific health challenges.

Given this background, the project was designed to develop, implement, and evaluate a CBE module on MCH for Bachelor of Science (BS) in Public Health students. It aims to address a critical gap in undergraduate public health education in Pakistan by offering a practical model that connects academic learning with real-world health challenges. While previous studies have highlighted the benefits of CBE broadly, this study specifically focuses on CBE in MNCH for undergraduate public health students. Prior research indicates that CBE can improve MCH indicators through direct community engagement, yet there is limited evidence on structured implementation and evaluation of such programs in higher education curricula [6,9].

Hands-on practice is critical for bridging theoretical knowledge with real-world skills, but practical training and systematic assessment remain underdeveloped in many public health programs in Pakistan. This study contributes to the literature by comprehensively evaluating a locally developed structured CBE module in MCH implemented through university-community partnership in a semi-urban context, with particular emphasis on the development of students’ knowledge, skills, and competencies within a well-designed program of study. While existing literature documents the value of CBE broadly, much of the empirical research has been conducted in in high-income countries or has focused on generalized health topics. The present study provides context-specific empirical evidence from a low- and middle- income country by examining how a structured CBE program can address priority MCH needs, build students practical skills, and strengthen community engagement. By assessing pre- and post-module changes in students’ knowledge, skills, and competencies, this study provides empirical evidence on the educational impact of CBE in a context where practical, community-focused training is largely absent. Thus, the study bridges a critical gap in both public health education and experiential learning in the region.

This paper specifically focuses on evaluating the pre and post assessment of CBE module in enhancing students’ knowledge, skills, and competencies in MCH. While the broader project also included assessment of community-level outcomes, those results are presented in a separate manuscript [10].

## Materials and methods

This paper is a part of a broader post-doctoral research project with International Foundation for Advancement of International Medical Education and Research (FAIMER) Institute, USA of RZ (first author) on the topic of “Improving Women’s Health and Strengthening CBE through Center for Research on Maternal and Child Health at Department of Public Health, University of the Punjab.” The project was conducted in three phases: First phase dealt with the selection of community, assessing the health needs of the selected community, development of CBE module on MCH, training of faculty and students on the CBE module, and pre-CBE implementation assessment of students’ knowledge, skills and competencies related to MCH (the results of community health need assessment [11] and module development [12] are published separately). The second phase was related to the implementation of the CBE module in the field, and the third phase aimed at evaluating its effectiveness from the perspective of both the students and the community. This paper specifically presents the findings of the impact of CBE on students’ MCH knowledge, skills, competencies, and learning experiences. A mixed-methods approach was used, with quantitative assessments conducted at pre- and post-implementation and qualitative assessments conducted during and after implementation. Evaluation methods included quizzes, MCQ examinations, pre- and post-tests, Objective Structured Clinical Examinations (OSCEs), focus group discussions (FGDs), and students’ self-reflection logs.

### First phase: Pre-implementation of CBE module‌‌

In the first phase, the selection of the study community was guided by the twelve-themed community selection matrix [13], a tool designed to identify communities with significant public health vulnerabilities and unmet needs. The community selection matrix was comprised of characteristics such as the number of beneficiaries, prevalent issues, accessibility, geographical attributes, historical events, communication means, existing programs, security/safety, community organization, and willingness. Each characteristic is scored, with a total possible score of 96. The community chosen for this study achieved a total score of 65, indicating notable vulnerabilities and making it a suitable setting for the intervention (for details see [11]). Following the selection of the semi-urban community, a baseline survey was conducted with mothers residing in the selected community to assess the MCH-related community health needs. The sampling frame included all households with mothers of reproductive age (15–49 years) having at least one child under five years of age. A systematic random sampling design was employed to ensure demographic and geographic representativeness. A total of 253 mothers and 371 children were surveyed and assessed as a part of the community health needs assessment (for further details see [11]). On the basis of baseline study findings, extensive review of relevant literature and curricula from different national and international universities, consultative meetings with faculty members and healthcare providers working in MCH domain, discussions with representatives of provincial health department, and need assessment of students, a CBE module was developed in MCH (for details see [12]).

### Second phase: Implementation of CBE module in the community

The CBE module was implemented in the second phase in the selected community by students of BS in Public Health program at the University of the Punjab over a one-year period (2^nd^ May 2023–30^th^ April 2024). As a pilot project, all (120) third-year students enrolled in the undergraduate Bachelor of Science (BS) program in Public Health were recruited over a one and a half year period (starting from 1^st^ Nov 2022). These students were involved in the module development from its inception, participating in community selection; baseline community health needs assessment, consultative meetings with faculty and healthcare providers, and the implementation and community evaluation of the module. The CBE module was integrated into the curriculum as part of the students’ internship program, making participation compulsory for all BS public health students [12].

All 120 third-year students of the BS Public Health program participated in the CBE implementation for 60 days, dedicating 4 hours per day over the course of one year. The students were divided into two shifts, morning and afternoon. Initially, a one-week training session was conducted by the lead researcher (RZ) for the faculty and research assistants responsible for delivering the CBE module in the community. Subsequently, the trained faculty provided a two-week training program for the students, covering all components of the CBE module. Various training modalities were employed, including lectures, simulations, mock exercises, and reflection sessions [12].

For community visits, each shift of students was further divided into four groups. Each group was supervised by a team of trained researchers and faculty in the community. The Lady Health Worker (LHW) (a trained female community health worker who delivers basic essential primary healthcare to a catchment population of 1500–2000 at the doorstep, especially in underserved areas) and the community social welfare worker assisted the students and faculty in connecting with the community. A research associate was assigned to each group to organize its learning activities in consultation with the lead researcher (RZ). Four faculty members were responsible for monitoring the students’ experiences during the CBE. At the beginning of each CBE session, students, together with the lead researcher and relevant faculty, identified their learning goals. Students were also expected to be punctual, polite, and respectful, communicate effectively, and work as part of a team.

During the implementation phase, students were provided training to screen for malnutrition among children, manage their therapeutic needs, assess their growth parameters, and conduct community consultation with mothers on lactation practices, family planning methods, hygiene and sanitation, child vaccination, and child development.

The students and community healthcare providers worked in small groups of 3–5 students. They regularly participated in mini-lectures with cases-based, problem-based learning. Students were encouraged to observe and interact with community members. In addition to participating in community awareness sessions, students were trained to provide education on safe birthing practices to traditional birth attendants and LHW from the community through interactive sessions, and the distribution of culturally appropriate educational materials.

During the community activities, students were required to fill out a logbook documenting their preparation time, the hours spent in the community, the nature of the services and activities performed, and the number of people served. Each community visit had to be verified by its group supervisor’s signature. Upon completing the CBE implementation, students prepared a report documenting their overall experiences, self-reflections, and the learning outcomes achieved through their community work.

Supervisors closely monitored the tasks of all students during training and fieldwork, conducted evaluations to assess their performance, and provided continuous feedback on their various learning activities. Regular reviews and reflections on field activities helped students enhance their communication skills.

### Third phase: Evaluation of the effectiveness of the CBE module

In the third phase, the effectiveness of CBE module was evaluated at both students’ and community level; however, this paper focuses on evaluation of CBE at students’ level.

#### Evaluation methods.

For the evaluation of the effectiveness of the CBE module, a multi-phased systematic evaluation approach was used employing both quantitative and qualitative methods. Data were collected at different points in time during the cycle of CBE implementation to evaluate the effectiveness of the CBE module on the learning outcomes of the students. This included data collection 1) at the pre-implementation phase, 2) during the implementation phase, and 3) at post-implementation phase.

#### Quantitative data.

Quantitative outcomes focused on three core domains of student learning: (1) cognitive outcomes (knowledge related to MCH), (2) affective outcomes (interpersonal and intrapersonal competencies), and (3) skill outcomes (practical skills assessed through OSCE). These domains guided the selection of assessment tools and statistical analyses conducted at pre-, during-, and post-implementation phases. The assessment was made by using both academic evaluation and research evaluation.

**Academic evaluation**: Academic evaluation was done to know students’ academic progress. For academic evaluation, the following methods were used:

- Students were assessed during and after the implementation of the CBE module on MCH knowledge through quizzes to know about their ongoing learning. Quizzes were administered at three points: (1) immediately after the training of students on the CBE module, (2) six months after its implementation, and (3) at the end of the CBE implementation. This approach helped in evaluating the ongoing learning of the students throughout the process.
- An end-of-module Multiple Choice Question (MCQ) exam focused on real-world MCH scenarios encountered during community visits to test student’s applied knowledge. This exam included 30 items covering antenatal care, immunization practices, malnutrition identification, and family planning.

**Research evaluation:** Research evaluation assessed the effectiveness of CBE through measuring outcomes, including changes in students’ knowledge, skills, and attitude related to MCH. For this, the following methods were used:

- Knowledge of students was assessed through pre- and post-tests on topics related to MCH.
- Assessment of students on their interpersonal and intrapersonal skills pre and post CBE module implementation.
- Skill development on MCH issues was measured using Objective Structured Clinical Examinations (OSCEs), consisted of six stations assessing students’ abilities in counseling for ANC, effective communication with caregivers, data collection from families, students’ competence in using MCH tools (e.g., growth charts), their ability to communicate health practices, such as hand washing technique, to mothers, and nutritional counseling for child health at the end of the CBE module implementation.

#### Qualitative data.

For gaining the qualitative insights into the learning experiences of the students and the impact of the CBE on their competencies, following methods were utilized:

- Qualitative interviews (FGDs) were conducted with selected students during the pre-implementation phase to understand their needs and expectations with CBE.
- Attitudinal changes were evaluated through self-reflection logs. Students submitted self-reflection narratives monthly during CBE implementation phase, which was primarily focused on empathy and cultural sensitivity. They described their narratives on their experiences, challenges, and learning.

Throughout the implementation, the lead researcher maintained a regular contact with the community focal person to assess the community’s satisfaction with the consultations and services provided by the students. Reflection and formative evaluation were integrated during the course of the implementation and events were arranged to celebrate the achievements of both students and community.

#### Sampling technique and sample size.

All 120 third-year students of BS Public Health program were engaged in the CBE implementation. However, in the final analysis only those students who completed one year of CBE program were included. All these students were approached for the collection of quantitative data for evaluation. For qualitative data, purposive sampling technique was used to ensure maximum variation of gender, field site, morning/evening shift, and academic performance (class grades/GPA). Four Focus group discussions (FGDs) were arranged with students, with 7–8 students in each group, making a total qualitative sample of 30 students. Similarly, reflection log books of all students who completed one-year CBE programs were included in the analysis.

#### Study tools.

For the collection of quantitative and qualitative data the following tools were used:

- For assessing the learning outcomes of the students (cognitive domain), three true/false quizzes were administered at different points in time: (1) after the training of the students on CBE module, (2) six months after implementation of the CBE module, and (3) at the completion of the CBE module implementation. Each correct response was awarded one mark, while incorrect responses received zero; higher total scores indicated better learning outcomes.
- To assess the applied knowledge of the students, a multiple-choice test was conducted. Each question provided four options with a single correct answer. Correct responses were awarded one mark, and incorrect responses scored zero. The total scores were then computed, with higher scores reflecting stronger applied knowledge among the students.
- For assessing the knowledge of students related to core MCH issues pre and post implementation of CBE module a structured questionnaire was used. It had a total of 14 questions regarding micro and macronutrients, 8 questions on personal hygiene practices and the consequences of poor hygiene, 16 questions about antenatal care (ANC) and its importance, 10 questions on family planning, 17 questions on intra-natal and post-natal care, 18 questions on malnutrition, 15 questions on breastfeeding, 10 questions on child immunization and vaccination, and 7 questions on psychomotor development. Each question was scored dichotomously, with a correct answer assigned a score of 1 and an incorrect answer assigned a score of 0. The mean scores for each domain were calculated.
- For measuring students’ inter and intra personal skills (affective domain) a self-rated questionnaire was used. Students were asked to self-rate their 13 inter-personal and intra-personal skills on a five-point Likert scale ranging from very poor to very good. The data collected at the pre-implementation stage served as the baseline data, while the same questionnaire was repeated at the post-implementation phase for evaluating the effectiveness of CBE implementation in strengthening the competencies of the students.
- To assess the skill development in the area of MCH (affective domain), a checklist was developed for OSCE according to the six stations; 1) students’ abilities in counseling for ANC, 2) effective communication with caregivers, 3) data collection from families, 4) students’ competence in using MCH tools (e.g., growth charts), 5) their ability to communicate health practices, such as hand washing technique to mothers, and 6) nutritional counseling for child health. Each station was scored with a maximum score of 10 points per station.
- For self-reflection, students submitted monthly written self-reflection log books during the CBE implementation focusing on their experiences in real community settings, specifically on empathy, respect for diverse cultural practices, and communication with underserved population. The logbooks were written in narrative form and treated as qualitative data for analysis.
- For understanding the needs and expectations of the students with CBE module at pre-implementation phase and their experiences at the post-implementation phase, a semi-structured FGD guide was used to conduct the discussions.

The summary of the type, methods, tools and purpose of evaluations used to assess the effectiveness of the CBE on MCH is illustrated in Table 1.

**Table 1:** Summary of type, method and purpose of evaluation used to assess the effectiveness of community-based education on maternal and child health.

Type of evaluation	Time/Phase	Method	Tools	Indicators	Purpose
Academic evaluation	In the second and third phase, during and at the end of implementation of CBE in the field	Quizzes	Paper-based true-false quizzes on MCH issues	Focuses on MCH issues	To know about students’ ongoing learning
	At the end of module implementation	MCQ exam	Paper based MCQs	Focused on real-world MCH scenarios	To test student’s applied knowledge
Research evaluation	In the first and third phase; pre and post implementation of CBE	Pre and post test	Structured questionnaire	On topics related to ANC, PNC, FP, immunization, breastfeeding and nutrition	To assess students’ knowledge on MCH issues
		Pre and post test	Structured questionnaire	Students’ inter and intra personal skills	To assess students’ inter and intra personal skills
	In the third phase, post implementation	Objective Structured Clinical Examinations (OSCEs)	checklist	Students’ abilities in counseling for ANC, communication with caregivers, data collection, competence in using MCH tools, communicate health practices, nutritional counseling	To assess students’ skill development on MCH issues
	In the second and third phase, during and post implementation	Self-reflection	Written self-reflection log books	focusing on empathy and cultural sensitivity and student’s learning experiences	To assess students’ attitude
	In the first and third phase, pre and post implementation	Focus group discussions (FGD) with students	FGD guide	Students’ needs and expectations and their experiences	Understand students’ needs and expectations and experiences with CBE

#### Ethical considerations.

The study was meticulously designed and conducted in strict adherence to ethical guidelines for epidemiological research. The research protocol underwent a comprehensive review and received approval from the Institutional Ethics Review Board (IERB) at University of the Punjab (No.D/358/FIMS). The study was conducted in accordance with ethical standards and the principles outlined in the Declaration of Helsinki. Written informed consent was obtained from all participants before their inclusion and confidentiality of data maintained throughout the study.

#### Data analysis.

For quantitative data analysis, we used IBM Statistical Package for Social Sciences (SPSS), Statistics for Windows version 26 [14]. Descriptive statistics (mean, standard deviation [SD], minimum, and maximum values) were calculated for quiz scores to evaluate students’ learning outcomes, as these measures provide a clear summary of central tendency and variability in performance. For MCQs, mean accuracy was calculated to quantify the proportion of correct responses, thereby serving as a direct indicator of knowledge acquisition. For the OSCE, mean scores (out of 10) along with SD were calculated, and the pass rate was determined using 55% as the passing score. The Marginal Homogeneity Test was applied to assess differences in students’ skills and competencies before and after CBE implementation. This test was selected as it is suitable for comparing paired categorical data across two time points, making it appropriate for measuring changes in students’ skills and competencies before and after CBE implementation. To compare students’ knowledge across different domains of MCH, the mean score in each domain was calculated, and pre- and post-implementation data were compared using a paired sample T-test. The paired t-test was chosen because it is appropriate for comparing means of normally distributed data obtained from the same participants at two different time points, thus allowing us to evaluate changes in knowledge attributable to the intervention

For the qualitative data of FGDs, thematic analysis was conducted using a deductive-inductive approach. Themes were developed based on predefined codes (deductive) derived from the CBE module, as well as emergent codes (inductive) identified from the data. The coding process was carried out manually by all the authors, to allow closer engagement with the transcripts and for a more nuanced, context-sensitive interpretation of responses. It helped researchers to immerse themselves in the data, capture subtle meanings, and account for cultural and contextual factors that software might not capture.

For self-reflection narratives, thematic analysis was conducted to explore their personal learning experiences. First, all the logbooks were collected and organized by month and student ID. The data from narratives were transcribed as most of the logs were handwritten by students. These were anonymized to ensure confidentiality of data. The two researchers read all reflections multiple times independently and got intimal impressions, identified recurring words and salient experiences. The researchers developed codes on the basis of key ideas, students’ feelings and experiences from the data. For example, “It improved confidence”, “difficulty managing time”, “community interaction skills”. The related codes were grouped into categories followed by broader themes.

An audit trial (including coding logs and decision records) was maintained for the thematic analysis of both, the FDGs and the self-reflection narratives, to enhance dependability and the analysis was reviewed by RZ and GS to ensure consistency and minimize personal bias. To ensure trustworthiness, credibility was supported through investigator triangulation and member checks, transferability through detailed contextual descriptions, dependability through the audit trail, and confirmability through reflexive note-taking. Discussions were held to resolve any disagreements between coders and it was ensured that the final themes accurately represented the data. Lastly, member checking was conducted, where the analysis was presented to a subset of the participants to ensure that the true meanings had been accurately extracted from their quotes.

Following this approach, the analysis of FGDs generated two main sets of themes: pre-CBE implementation (students’ needs and expectations, e.g., fieldwork exposure, application of theory, and skill development) and post-CBE implementation (students’ experiences, e.g., applying knowledge in real-world contexts, community engagement, professional readiness, and personal growth). The analysis of self-reflection logs revealed recurring themes of professionalism, teamwork, empathy, respect for cultural beliefs, and recognition of contextual challenges in community health. The frequency of these themes (e.g., empathy and cultural respect appearing in the majority of logs) further supported the credibility of findings by triangulating interview data with students’ own reflective accounts.

## Results

Out of 120 students, 102 students completed all the activities over the period of 2 years. About 18 students were skipped because of ailment or drop out due to their personal reasons.

### Pre-CBE module implementation: Students’ needs and expectations

The qualitative findings from the needs assessment for CBE highlighted several fundamental needs among students. They indicated a lack of practical fieldwork experience, an understanding of real-world public health issues, knowledge of MCH needs, and familiarity with community needs and engagement. Students expressed confusion about applying theoretical knowledge to real-world situations and emphasized the need for more knowledge on measuring anthropometric information. As one student mentioned, *“We have completed half of our degree program, but we are very confused about how this knowledge is applicable to real-life situations*.” Another student stated, *“I am afraid that I will not be able to deliver what I have learned when I start my career because I do not have any practical experience.”* Similarly, another student said, *“I do not know what we will do after completing this degree because we have no experience with fieldwork. We do not know how to work in communities*.”

Additionally, some students pointed out that the current curriculum should address implementing research design through various field activities focusing on data collection processes, probing techniques, and taking field notes. One student highlighted, *“We have studied research methodology courses, but we do not know how to implement them practically.*” Furthermore, students mentioned several interpersonal and intrapersonal skills they expected to develop through the CBE, including communication skills, the ability to design tailored health education and promotion campaigns, an understanding of field ethics, and the ability to handle field challenges and unexpected situations.

Students also shared their expectations for CBE, hoping the module would address their perceived deficiencies and prepare them professionally for the future. One student mentioned*, “All our teachers tell us that public health experts work in communities and not in clinical settings, but they do not provide us exposure to communities and real-world issues.”* Another student said, *“We expect a comprehensive program that not only equips us with personal skills but also public health and research skills.”* Highlighting the differing needs of communities, one student stated, *“I belong to a very backward village where we do not have proper hygienic facilities, but in Lahore (a developed urban city), the scenario is very different, and the issues are very different. How can we tackle and address these different issues in different ways?”*

### Quantitative findings

This section is divided into the results of academic and research evaluations.

#### Academic evaluation: To assess students’ ongoing learning.

**Quiz scores.** During the implementation of the CBE module on MCH, students were assessed through quizzes to know about their ongoing learning. The mean quiz score across all participants was 80.2% (SD = 7.2), with scores ranging from 60% to 96% (Table 2).

**Table 2:** Quiz score of students on maternal and child health issues during CBE module implementation.

Quiz number	Mean score (%)	SD (Standard deviation)	Min score	Max score
Quiz 1 (after the training sessions on CBE module)	75.4	7.2	60	90
Quiz 2 (6 months after the module implementation)	78.1	9.1	62	94
Quiz 3 (at the end of the implementation)	87.0	5.3	75	96

**MCQs based on real community settings.** The mean MCQ exam score was 81.5% (SD = 7.6), with 85% of students scoring above 75%. Questions based on case scenarios in immunization and antenatal care had the highest accuracy rates (92% and 89% respectively), while questions on growth monitoring had lower scores (65%) (S1 Fig).

### Research Evaluation: Pre and post analysis of students’ knowledge in maternal and child health issues to assess the effectiveness of CBE

The comparison of students’ knowledge before and after the CBE implementation showed significant improvements. The mean scores for knowledge on micro and macronutrients increased from 5.4 ± 2.5 to 9.2 ± 2.1 (p < 0.001; t = −21.1; d = −2.1); personal hygiene practices from 3.0 ± 1.5 to 5.6 ± 1.1 (p < 0.001; t = −19.3; d = −1.9); ANC from 5.9 ± 2.8 to 10.3 ± 2.1 (p < 0.001; t = −27.8; d = −2.8); family planning from 3.6 ± 1.9 to 7.5 ± 1.2 (p < 0.001; t = −30.2; d = −3.0); intra-natal and post-natal care from 6.4 ± 3.1 to 11.1 ± 2.3 (p < 0.001; t = −30.9; d = −3.1); malnutrition from 6.7 ± 3.2 to 10.0 ± 2.7 (p < 0.001; t = −19.0; d = −1.9); breastfeeding practices from 5.5 ± 2.6 to 9.2 ± 1.8 (p < 0.001; t = −24.9; d = −2.5); child immunization and vaccination from 3.7 ± 1.8 to 6.7 ± 1.3 (p < 0.001; t = −23.2; d = −2.3); and psychomotor development from 2.5 ± 1.2 to 5.3 ± 0.8 (p < 0.001; t = −22.0; d = −2.2) after CBE implementation (Table 3).

**Table 3:** Comparison of student knowledge on maternal and child health issues pre- and post-community based education module implementation.

Maternal and child health issues	Pre-CBE implementation (n = 102)	Post-CBE implementation (n = 102)	p-value*	t-value	Cohen’s d value
	mean±SD	mean±SD			
Information on micro- and macro nutrients, daily recommended intake, energy requirement by age and lifestyle. Information on personal hygiene practices, consequences of poor hygiene, and control measures to prevent disease transmission	5.4 ± 2.5	9.2 ± 2.1	<0.001	−21.1	−2.1
Understanding the concept, importance and components of personal hygiene, identify common practices that promote hygiene, recognize the relationship between hygiene and health outcomes, and demonstrate proper handwashing and different hygiene techniques	3.0 ± 1.5	5.6 ± 1.1	<0.001	−19.3	−1.9
Understanding the concept of ANC and its importance, identify signs of anemia through physical examination of women, identify warning signs of pregnancy among women, define the importance of health education and counseling in promoting healthy behaviors	5.9 ± 2.8	10.3 ± 2.1	<0.001	−27.8	−2.8
Address the need and importance of family planning, distinguish between different types of family planning methods and identify barriers to access and barriers to utilization of family planning methods in different communities	3.6 ± 1.9	7.5 ± 1.2	<0.001	−30.2	−3.0
Distinguish between intra-natal and post-natal care and discuss their importance and identify post-natal mother and newborn danger signs	6.4 ± 3.1	11.1 ± 2.3	<0.001	−30.9	−3.1
Distinguish between different types of malnutrition among children [Identify severe acute malnutrition (SAM), moderate acute malnutrition (MAM), and at risk of malnutrition children by taking anthropometric measures]	6.7 ± 3.2	10.0 ± 2.7	<0.001	−19.0	−1.9
Discuss nutritional requirement in childhood and the importance of breast milk, the importance of early initiation of breastfeeding, identify the major breastfeeding difficulties and define their management strategies	5.5 ± 2.6	9.2 ± 1.8	<0.001	−24.9	−2.5
Define the concept and importance of timely child immunization and different vaccinations, their need and side-effects	3.7 ± 1.8	6.7 ± 1.3	<0.001	−23.2	−2.3
Define the concept of psychomotor development and differentiate between different psychomotor developmental skills	2.5 ± 1.2	5.3 ± 0.9	<0.001	−22.0	−2.2

Abbreviations: SD, Standard Deviation; ANC, Antenatal Care; SAM, Severe Acute Malnutrition; MAM, Moderate Acute Malnutrition; CBE, Community-Based Education.

*: Indicates paired sample t-test was used at 95% confidence interval.

### Pre and post analysis of students’ interpersonal and intrapersonal skills

Before engagement in the CBE, a majority of students demonstrated poor skills in leadership (very poor = 16.7%; poor = 37.3%), project management (very poor = 10.8%; poor = 38.2%), time management (very poor = 15.7%; poor = 35.3%), communication (very poor = 13.7%; poor = 35.3%), and problem-solving (very poor = 7.8%; poor = 38.2%). After a year of involvement in the CBE, these skills showed significant improvement, with most students demonstrating above-average abilities: leadership (p < 0.001), project management (p < 0.001), time management (p < 0.001), communication (p < 0.001), and problem-solving (p < 0.001) (Table 5).

Additional skills also showed significant improvement, including the ability to handle unexpected situations (p < 0.001), work productively in a team (p <<0.001), identify community needs (p < 0.001), design tailored interventions (p <<0.001), and transfer theoretical knowledge into practical applications (p < 0.001). The students’ understanding of real public health issues significantly improved, with only 7.8% demonstrating good understanding before CBE, compared to over 90% showing better understanding at the end (p < 0.001). Similarly, there was a significant improvement in their understanding of the intersectionality of public health issues (p < 0.001) and social responsibility (9.8% good and 1.0% very good pre-CBE to 32.5% good and 56.9% very good post-CBE (Table 4).

**Table 4:** Comparison of students’ interpersonal and intrapersonal skills pre- and post-community based education module implementation (n = 102).

Inter and intra Personal Skills of Students	Pre-CBE		Post-CBE		test value*	p-value
	f	%	f	%		
**Leadership Skills**						
Very Poor	17	16.7	1	1.0	−8.9	<0.001
Poor	38	37.3	1	1.0		
Average	39	38.2	1	1.0		
Good	8	7.8	66	64.7		
Very Good	0	0.0	33	32.4		
**Project management skills**						
Very Poor	11	10.8	0	0.0	−8.9	<0.001
Poor	39	38.2	3	2.9		
Average	42	41.2	5	4.9		
Good	10	9.8	55	53.9		
Very Good	0	0.0	39	38.2		
**Time Management Skills**						
Very Poor	16	15.7	1	1.0	−8.7	<0.001
Poor	36	35.3	0	0.0		
Average	40	39.2	12	11.8		
Good	10	9.8	36	35.3		
Very Good	0	0.0	53	52.0		
**Communication Skills**						
Very Poor	14	13.7	0	0.0	−8.5	<0.001
Poor	36	35.3	3	2.9		
Average	41	40.2	9	8.8		
Good	10	9.8	46	45.1		
Very Good	1	1.0	44	43.1		
**Problem Solving Skills**						
Very Poor	8	7.8	0	0.0	−8.3	<0.001
Poor	39	38.2	1	1.0		
Average	40	39.2	16	15.7		
Good	15	14.7	47	46.1		
Very Good	0	0.0	38	37.3		
**Skills to deal with unexpected situations**						
Very Poor	5	4.9	1	1.0	−8.745	<0.001
Poor	53	52.0	1	1.0		
Average	38	37.3	12	11.8		
Good	6	5.9	51	50.0		
Very Good	0	0.0	37	36.3		
**Skills to work productively in team**						
Very Poor	11	10.8	0	0.0	−9.1	<0.001
Poor	48	47.1	1	1.0		
Average	38	37.3	11	10.8		
Good	5	4.9	39	38.2		
Very Good	0	0.0	51	50.0		
**Understanding of real issues pertaining to public health**						
Very Poor	10	9.8	0	0.0	−8.9	<0.001
Poor	35	34.3	2	2.0		
Average	49	48.0	6	5.9		
Good	8	7.8	51	50.0		
Very Good	0	0.0	43	42.2		
**Understanding of intersectionality of public health issues**						
Very Poor	16	15.7	0	0.0	−8.8	<0.001
Poor	47	46.1	1	1.0		
Average	33	32.4	13	12.7		
Good	6	5.9	50	49.0		
Very Good	0	0.0	38	37.3		
**Skills to design tailored interventions according to the needs of community**						
Very Poor	16	15.7	0	0.0	−8.8	<0.001
Poor	44	43.1	2	2.0		
Average	34	33.3	10	9.8		
Good	8	7.8	55	53.9		
Very Good	0	0.0	35	34.3		
**Skills to identify the needs of community**						
Very Poor	14	13.7	0	0.0	−8.8	<0.001
Poor	45	44.1	2	2.0		
Average	34	33.3	8	7.8		
Good	9	8.8	35	34.3		
Very Good	0	0.0	57	55.9		
**Understanding of one’s social responsibility**						
Very Poor	12	11.8	0	0.0	−8.7	<0.001
Poor	43	42.2	3	2.9		
Average	36	35.3	8	7.8		
Good	10	9.8	33	32.4		
Very Good	1	1.0	58	56.9		
**Skills to transfer theoretical knowledge to practical knowledge**						
Very Poor	13	12.7	0	0.0	−9.1	<0.001
Poor	45	44.1	1	1.0		
Average	37	36.3	8	7.8		
Good	7	6.9	45	44.1		
Very Good	0	0.0	48	47.1		

Abbreviations: CBE, Community-Based Education; f, Frequency (number of students).

*: Indicates Marginal Homogeneity Test was used

### Results of objective structured clinical examination (OSCE)

Students performed well in communication (mean = 8.5) and hand hygiene demonstrations (mean = 8.7), indicating strong applied skills in these areas (Table 4). However, challenges were observed in interpreting growth charts (mean = 7.1), suggesting the need for further practical reinforcement (Table 5).

**Table 5:** Station-wise performance OSCE score.

OSCE Station	Mean score (out of 10)	SD	Pass rate (%)
Antenatal Counseling	8.2	1.1	91
Communication with Mother	8.5	0.9	94
Data Collection from Family	7.9	1.4	88
Use of Growth Chart	7.1	1.8	76
Explaining Hand-washing Techniques	8.7	0.8	96
Nutritional Counseling for Child Health	7.5	1.6	82

SD: standard deviation.

OSCS: Objective Structured Clinical Examination.

### Findings from self-refection

Self-reflection helped students to examine their own thoughts, feelings, behaviors and experiences to gain deeper understanding of themselves, their actions, and their impact. They assessed what went well, what could be improved, and how to apply lessons learned for their professional growth. The thematic analysis of students’ reflection logs identified that a majority of students demonstrated high levels of professionalism, effective teamwork, and strong respect for others. Faculty observations corroborated students’ reflections, indicating respectful engagement with community members and effective peer collaboration during field activities. Out of 102 students, 86 (86%) showed high levels of empathetic engagement in their reflections, and 78 (77%) explicitly discussed adjusting their approach due to cultural beliefs. Thematic frequency analysis revealed “empathy” appeared in 91% of logs, and majority of the students shared that they recognized the emotional struggles of pregnant women in poor communities. One of the students opined: *“I realized how just listening and showing concern can comfort a mother.”*

Many students believed that through such activities (referring to CBE) their awareness of community’s religious and health beliefs improved. Thematic frequency analysis shown “respect for culture” was mentioned in 82% of logs. One student shared: “while communicating with mothers, I had to adjust my language and tone to align with community’s norms, so they don’t feel that I am an outsider”. Similarly, students acknowledged their misconceptions regarding community’s unhealthy behaviors. One student said: “I used to think people did not care about health, but I saw their struggles firsthand. I realized, the environment and the socio-economic situation they are living in is a great challenge for them to maintain their health.”

### Post-CBE module implementation: Students’ experience

Findings from qualitative interviews with students’ post-CBE implementation revealed that CBE provided them with a unique, challenging, yet positive real-world experience that equipped them with various skills through direct community engagement. Students mentioned that they had complete theoretical knowledge regarding public health, but the applicability of this knowledge was lacking. However, when they interacted with the community, they encountered a completely different domain of real-world public health issues, giving them a new experience and perspective. One student mentioned, “Working directly with the community gave me a new perspective on the challenges people face and how I can use my skills to make a real difference.”

Students emphasized that CBE was a transformative experience, providing opportunities to connect with the community, develop various skills, and apply theoretical knowledge to real-world situations. One student said, “This experience has been transformative. I feel more connected to my community and more confident in my ability to effect change.” Another added, “Applying what I’ve learned in the classroom to real-world situations has been a wonderful experience. It has made my degree and education feels much more relevant.” Despite the field challenges, the positive health outcomes and the trust relationship with the community encouraged students to continue working for the community. One student mentioned, “This module was the highlight of my academic journey. It gave me hands-on experience and allowed me to see the tangible outcomes of my work. Although we faced many challenges initially because it was our first experience, it was heartwarming to see how mothers and their children trust us.”

Moreover, students highlighted that their engagement in CBE helped them polish their interpersonal and intrapersonal skills. One student mentioned, “I used to think that I had very good communication skills, but during my first field experience, I realized my shortcomings, and CBE completely transformed my personal skills.” Another student added, “Being able to work with community members taught me empathy and the importance of listening. It was a humbling experience.” Students noted that their exposure and engagement with different community members not only enhanced their skills but also provided opportunities for mutual learning and understanding the societal issues underpinning the broader public health domain. One student said, “The relationships I built with community partners and fellow students were invaluable. We learned so much from each other.” Another student mentioned, “I never realized how much I could learn outside the classroom. This experience has broadened my perspective and deepened my understanding of societal issues.” Another added, “This experience has taught me to think critically and creatively. It has pushed me out of my comfort zone and helped me grow both personally and professionally.”

Overall, CBE offered valuable experiential learning opportunities to students, enhancing their academic skills and fostering personal development. While certain challenges were present, the benefits outweighed the difficulties, making CBE a worthwhile component of the educational program.

## Discussion

This study aimed to implement and evaluate the CBE module in MCH for public health students through a community outreach program. The module offered practical field teaching and community-based education to Bachelor of Science students from the disciplines of Public Health. This initiative fostered a robust community-university partnership by enhancing the skills, competencies, and knowledge of students in MCH and social determinants of health. The implementation of the CBE module by university marked a significant step in applying theoretical knowledge to practical settings. Over two years, 120 third-year public health students were engaged in developing and deploying the module, which included direct engagement in community assessments and educational activities. Given the persistent underutilization of MCH services in Pakistan, particularly in semi-urban and rural areas [6,15], strengthening the competencies of future public health professionals in MCH through CBE is both timely and essential. Previous studies have highlighted that preventive strategies are often focused on supply side factors (related to availability, accessibility, and quality of healthcare services) through the revamping of existing healthcare facilities. However, the demand-side factors such as community mobilization and awareness regarding healthcare needs and health conditions remain neglected [16]. Given this context, community-based initiatives, such as behavior change interventions targeting mothers and caregivers, delivered through the involvement of university students can be particularly beneficial.

The CBE module demonstrated substantial promise based on our findings. The pre- and post-intervention data indicated significant improvements in students’ knowledge, skills and competencies in MCH issues, as well as their leadership, project management, communication, and teamwork, and problem-solving skills. Literature also indicates the development of certain soft skills (non-technical) among students through community engagement services that employers increasingly require from students entering the job market [17].

The results of the present study align with previous findings, highlighting that community engagement enhances students’ life-skills by increasing their awareness of social responsibility, and their ability to work in diverse teams, multi-disciplinary environments and unexpected outcomes [3,18,19]. However, some challenges emerged that warrant critical reflection. Notably, relatively lower scores were observed among students in the domain of growth monitoring and malnutrition management. This may be attributed to limited emphasis during training, complexity of the content, or contextual barriers such as lack of community resources and variability in children’s nutritional status. These findings highlight the need for strengthening content delivery in these areas in future iterations of the module. Unanticipated challenges also emerged during implementation. For instance, variations in community participation were observed, including fluctuating attendance at health education sessions, differential engagement of households due to work schedules and gender norms, and occasional reluctance among some families to participate in student-led consultations. These factors influenced the intensity and continuity of student-community interactions during the intervention. Other challenges included logistical constraints in semi-urban fieldwork, and occasional difficulties in sustaining student motivation presented barriers. While these challenges did not undermine the feasibility of the intervention, they point to the importance of building more structured support systems for both students and communities.

The findings of present research indicate that this hands-on approach not only facilitated real-world learning but also strengthened university-community partnership. Consistent with previous research, community-oriented teaching provided students with opportunities to learn about the health needs and demands of the population they will serve [3,19]. Additionally, students were able to get deeper understanding of social, political and economic factors affecting health, known as social determinants of health [3,20,21]. The dual focus of the CBE module on community health and student competency development underscores its crucial role in enhancing educational quality and public health outcomes. This comprehensive approach ensured that the positive impacts of academic programs extend beyond the classroom, which resulted in significant community health improvements and the enrichment of students’ skills and capacities. The project effectively bridged the gap between academic learning and practical application, while also establishing a sustainable partnership between the university and the community, setting a valuable precedent for future public health initiatives [1].

The results are promising, however, the challenge remains in sustaining these outcomes and the scalability and adaptability of the module warrant consideration. The positive feedback from students regarding the CBE module suggests that integrating such modules into regular academic curricula could enhance practical skills and awareness among future health professionals. This could also foster a stronger connection between academic institutions and community health needs.. Nonetheless, the framework offered a model that can be adapted in other regions, particularly where community-based learning is underutilized.

This study, the first of its kind in Punjab, has several limitations. Firstly, no a priori power analysis was conducted, given the evaluative and formative nature of the study; this limits statistical generalizability. Further, the contextual differences, such as variations in health systems, community engagement, and educational priorities, may also affect generalizability. Secondly, we did not collect data on students’ socio-demographic characteristics, preventing subgroup or stratified analyses (e.g., by gender, prior MCH exposure, or academic performance), though these may reveal heterogeneity in outcomes and should be explored in future research. Third, potential biases such as social desirability bias in self-assessments and observer bias during skills evaluation cannot be fully excluded. Further, the study was limited to a single university, which may constrain external validity. Additionally, there were practical challenges, such as securing resources (e.g., funds for students’ travel to the community), limited community exposure for students, and the diverse population within the community.

Despite these challenges and limitations, the study contributes to understanding how CBE can bridge the gap between academic learning and practice. Continuous funding, government support, and sustained community engagement are essential for long-term success. Integration of such modules into regular curricula, coupled with ongoing evaluation and adaptation, could further strengthen public health education. Future research may also explore digital innovations to enhance module delivery, scalability, and impact.

## Practice points

- The CBE module in maternal and child health was highly valued by both students and faculty.
- Community-based teaching offers students a transformative learning experience.
- The CBE module engages students with the community, helping them understand specific needs, challenges, and social determinants of health.
- Sustaining a CBE program requires faculty training, collaboration with health and developmental organizations, and strong community support.

## Conclusions

The CBE module implemented at the University of the Punjab has significantly enhanced educational outcomes by effectively bridging theoretical knowledge with practical application. This initiative provided students with invaluable real-world public health experience while simultaneously addressing critical health needs within the community. Through structured training and direct community engagement, students developed essential skills and competencies crucial for effective public health practice. The project demonstrated the potential of university-community partnerships to foster meaningful and sustainable changes in public health infrastructure.

In conclusion, the CBE module enhanced the educational journey of public health students by improving their learning and professional development, showcasing the power of collaborative efforts in advancing public health goals. These outcomes affirm the value of integrating community-based education into public health curricula, suggesting a robust model for other institutions to follow.

Future research should focus on longitudinal studies to track the long-term impact of CBE modules on both student competencies and community health outcomes. Additionally, exploring the scalability and adaptability of the CBE model in different geographic and cultural contexts could provide valuable insights for broader implementation. There is an urgent need to strengthen university-community partnerships, increase investment in faculty training, and continuously refine CBE curricula based on feedback and evolving public health challenges.

## Supporting information

S1 Fig Multiple choice question exam score of students on maternal and child health related topics. (DOCX)

S2 File SPSS dataset used for analysis. (ZIP)
