# Supplementary material for: Empowering future public health professionals: The impact of community-based education on students’ maternal and child health competencies
Source: PLoS One. 2026 Jun 1;21(6):e0345916. doi: 10.1371/journal.pone.0345916 (PMC13225399; doi:10.1371/journal.pone.0345916)
Supplement: S1 Fig — (DOCX) [file pone.0345916.s001.docx]

S1 Fig 1: Multiple-choice question exam score of students on maternal and child health-related topics
